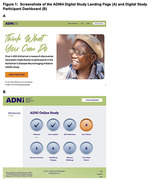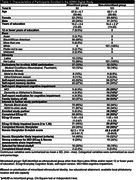# The ADNI4 Digital Study: A novel approach to recruitment, screening, and assessment of participants for AD clinical research

**DOI:** 10.1002/alz.091681

**Published:** 2025-01-09

**Authors:** Rachel L. Nosheny, Melanie J. Miller, Bruce Albala, Catherine C. Conti, Derek Flenniken, Juliet Fockler, Winnie Kwang, Diana Truran‐Sacrey, Adam Diaz, Miriam T. Ashford, Caroline Skirrow, Jack Weston, Emil Fristed, Sarah Tomaszewski Farias, Magdalena Korecka, Wan Yang, Eddie B Lee, Paul S. S. Aisen, Ronald C. Petersen, Leslie M. Shaw, Ozioma C Okonkwo, Monica Rivera Mindt, Michael S. W. Weiner

**Affiliations:** ^1^ University of California, San Francisco, San Francisco, CA USA; ^2^ San Francisco Veterans Affairs Medical Center, San Francisco, CA USA; ^3^ Northern California Institute for Research and Education (NCIRE), San Francisco, CA USA; ^4^ University of California Irvine, Irvine, CA USA; ^5^ Veterans Affairs Medical Center, San Francisco, CA USA; ^6^ Novoic, London United Kingdom; ^7^ University of California, Davis, Sacramento, CA USA; ^8^ Perelman School of Medicine, University of Pennsylvania, Department of Pathology and Laboratory Medicine, Philadelphia, PA USA; ^9^ University of Pennsylvania, Philadelphia, PA USA; ^10^ Perelman School of Medicine at the University of Pennsylvania, Philadelphia, PA USA; ^11^ Alzheimer’s Therapeutic Research Institute, San Diego, CA USA; ^12^ Mayo Clinic Alzheimer’s Disease Research Center, Rochester, MN USA; ^13^ University of Wisconsin, Madison, WI USA; ^14^ Icahn School of Medicine at Mount Sinai, New York, NY USA; ^15^ Fordham University, New York, NY USA

## Abstract

**Background:**

Scalable, efficient methods are needed to enroll diverse populations of older adults into AD observational studies and clinical trials. We evaluated preliminary feasibility of a novel, digital, culturally informed approach to recruit and screen participants for the Alzheimer’s Disease Neuroimaging Initiative (ADNI4).

**Methods:**

Digital advertising tailored towards Black/African American and Latinx older adults residing near six clinical ADNI sites directed potential participants to a recruitment website (**Figure 1**). They completed digital surveys of demographics, ADNI exclusion criteria, memory concerns and changes, self‐report cognitive impairment, and the Everyday Cognition Scale (ECog)‐12 item. Participants also completed Novoic Storyteller, a self‐administered speech‐based cognitive test. Digital assessment results were used to prioritize those from minoritized ethnocultural groups, with low educational attainment, and possible Mild Cognitive Impairment (MCI) for remote blood draw at local Quest phlebotomy centers to obtain AD plasma biomarkers. Completion rates and digital assessment performance were compared between minoritized and non‐minoritized groups.

**Results:**

Of 989 participants recruited from digital advertising efforts who provided contact information, 230 (23%) signed consent, and 183 (19%) completed at least one remote assessment (**Table 1**). 45% were from minoritized ethnocultural groups, 4% had ≤12 years of education, and 53% had evidence of possible MCI from digital assessments. 34 (19%) were referred to the plasma biomarker study, of which 21% completed blood draw. Compared to non‐minoritized participants, minoritized participants were less likely to report a diagnosed cognitive impairment or a family history of AD, less likely to have ECog scores indicative of MCI, and less likely to have a study partner. Minoritized participants had lower ECog and Novoic Storyteller completion rates.

**Conclusion:**

Recruitment and assessment of a diverse cohort of older adults, including those with possible cognitive impairment, is feasible using culturally informed digital advertising. Improving study engagement and achieving educational diversity are key challenges. This approach is now being scaled up to facilitate recruitment into in‐clinic ADNI4, with the goal of enrolling 500 new participants: >50% from minoritized groups, 40% with MCI, and 80% amyloid positive across diagnostic groups. This approach can be adapted to facilitate recruitment and longitudinal assessment in other AD studies and trials.